# Oxygen and Air Nanobubble Water Solution Promote the Growth of Plants, Fishes, and Mice

**DOI:** 10.1371/journal.pone.0065339

**Published:** 2013-06-05

**Authors:** Kosuke Ebina, Kenrin Shi, Makoto Hirao, Jun Hashimoto, Yoshitaka Kawato, Shoichi Kaneshiro, Tokimitsu Morimoto, Kota Koizumi, Hideki Yoshikawa

**Affiliations:** 1 Department of Orthopaedic Surgery, Graduate School of Medicine, Osaka University, Suita, Osaka, Japan; 2 Department of Orthopaedic Surgery, National Hospital Organization, Osaka Minami Medical Center, Kawachinagano, Osaka, Japan; 3 Department of Immunology, National Hospital Organization, Osaka Minami Medical Center, Kawachinagano, Osaka, Japan; Catalan Institute for Water Research (ICRA), Spain

## Abstract

Nanobubbles (<200 nm in diameter) have several unique properties such as long lifetime in liquid owing to its negatively charged surface, and its high gas solubility into the liquid owing to its high internal pressure. They are used in variety of fields including diagnostic aids and drug delivery, while there are no reports assessing their effects on the growth of lives. Nanobubbles of air or oxygen gas were generated using a nanobubble aerator (BUVITAS; Ligaric Company Limited, Osaka, Japan). Brassica campestris were cultured hydroponically for 4 weeks within air-nanobubble water or within normal water. Sweetfish (for 3 weeks) and rainbow trout (for 6 weeks) were kept either within air-nanobubble water or within normal water. Finally, 5 week-old male DBA1/J mice were bred with normal free-chaw and free-drinking either of oxygen-nanobubble water or of normal water for 12 weeks. Oxygen-nanobubble significantly increased the dissolved oxygen concentration of water as well as concentration/size of nanobubbles which were relatively stable for 70 days. Air-nanobubble water significantly promoted the height (19.1 vs. 16.7 cm; P<0.05), length of leaves (24.4 vs. 22.4 cm; P<0.01), and aerial fresh weight (27.3 vs. 20.3 g; P<0.01) of Brassica campestris compared to normal water. Total weight of sweetfish increased from 3.0 to 6.4 kg in normal water, whereas it increased from 3.0 to 10.2 kg in air-nanobubble water. In addition, total weight of rainbow trout increased from 50.0 to 129.5 kg in normal water, whereas it increased from 50.0 to 148.0 kg in air-nanobubble water. Free oral intake of oxygen-nanobubble water significantly promoted the weight (23.5 vs. 21.8 g; P<0.01) and the length (17.0 vs. 16.1 cm; P<0.001) of mice compared to that of normal water. We have demonstrated for the first time that oxygen and air-nanobubble water may be potentially effective tools for the growth of lives.

## Introduction

Nanobubbles are miniature gas bubbles in liquids with <200 nm in diameter, and have several unique physical properties [1]. They remain stable in water for a long time because of their negatively charged surface (zeta potential), whereas macrobubbles increase in size, rise rapidly and burst at the water surface [2], [3]. In addition, internal pressure of nanobubbles in liquids is much higher than that of their environment, which accelerates dissolution of the gas into the liquids [4], [5]. This remarkable property of nanobubbles, highly efficient gas solubility, was previously reported in super-saturation of oxygen gas in water [1], [3]. The smaller the bubble size, the higher the oxygen pressure (PO_2_) values in water [6], suggesting that nannobubbles increase the PO_2_ values in water to greater extent than that of microbubbles (10–50 µm in diameter) [1], [2]. It has been reported that high oxygen gas solubility of microbubbles is beneficial for oxygenation of hypoxic tissues [7], [8], [9], and their variable applications for medicine are expected to be useful [10], [11], [12]. Very recent *ex vivo* study has demonstrated that oxygen-nanobubble saline effectively improved hypoxic conditions of swine blood [2].

Previous studies have demonstrated that hyperoxia promotes the growth of plants [13] and animals [14], from which we assumed that air and oxygen-nanobubbles may affect the growth of life by changing oxygen condition. Indeed, a previous study showed that air-microbubbles promoted the growth of leaf lettuce compared to air-macrobubbles [15], but it was the study only on plants, not on animals. Moreover, no study of the effect of fine nanobubbles on the growth of lives have so far been reported.

In this paper, we studied whether air and oxygen-nanobubble water can be safely used and can affect the growth of plants, fishes, and mammals.

## Materials and Methods

### Ethics Statement

The animal experimental protocol was approved by the ethics review committee for animal experimentation of Osaka University School of Medicine.

**Figure 1:**
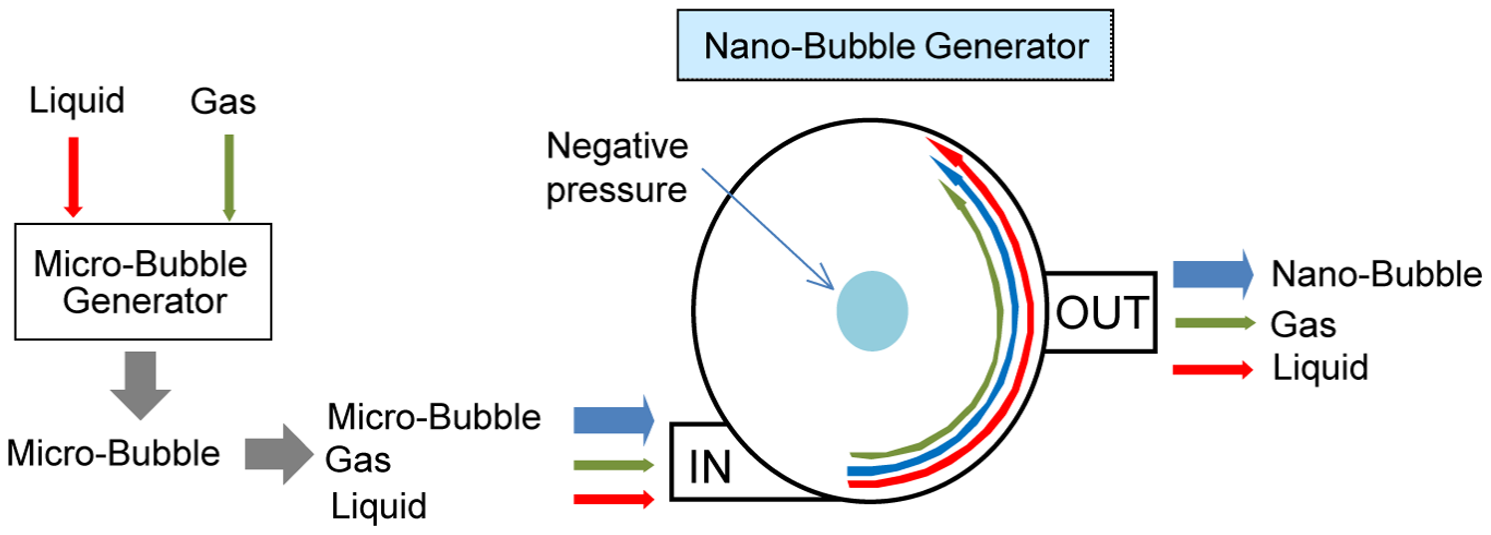
Nanobubble aerator (BUVITAS; Ligaric Company Limited, Osaka, Japan). Fine microbubbles of gas were generated first after brief sonication. Then nanobubbles were generated using this gas-liquid mixing system with hydrodynamic function. In this apparatus, gas was supplied at 0.1 MPa and 0.7 L/min into microbubble water for 30 min. The high-speed centrifugal force caused by the circulation separates the microbubbles into fine nanobubbles by the strong shearing force in the dispersed water.

Fine microbubbles of gas were generated first after brief sonication. Then nanobubbles were generated using a nanobubble aerator (BUVITAS; Ligaric Company Limited, Osaka, Japan), gas-liquid mixing system with hydrodynamic function (Figure 1). In this apparatus, gas was supplied at 0.1 MPa and 0.7 L/min into microbubbles water for 30 min, which is introduced by a pump (3600 r.p.m.), spirals along the inner wall and finally goes down to the outlet. The high-speed centrifugal force caused by the circulation separates the microbubbles into fine nanobubbles by the strong shearing force in the dispersed water.

Dissolved oxygen concentration (DO) of oxygen-nanobubble water during generation by BUVITAS was measured sequentially by Winkler's method [16]. To confirm the sequential change of number and diameter of generated air-nanobubbles, water containing air-nanobubbles was morphologically analyzed by Multisizer 3 (Beckman Coulter, Inc., Miami, FL, USA) [17].

Brassica campestris (32 stumps in each group) were cultured hydroponically for 4 weeks within air-nanobubble water or within normal water. Water was kept circulated (38 L/min) in the tank (125 L) and air-nanobubble solution was continuously generated using the nanobubble aerator. The water level of the tank and water temperature (from 23.2 to 24.3°C was maintained throughout the culture. The height, length of leaves, and aerial fresh weight of plants were monitored after 4 weeks. In addition, DO (by Winkler's method), pH, and other growth-affecting elements (nitrogen, phosphorus, potassium, calcium, and magnesium) of cultured water were also monitored sequentially. To assess the effect of air-nanobubbles on the growth of fishes, sweetfish (for 3 weeks) and rainbow trout (for 6 weeks) were kept either within air-nanobubble water (nanobubble aerator was always kept working to circulate 67 L air-nanobubble water/min) or within normal water. Normal fishbait was provided by 13% of primary total weight per day in each group. Finally, to assess the effect of oxygen-nanobubbles on the growth of mice, 5 week-old male DBA1/J mice were bred for 12 weeks with free-normal chaw (Oriental Yeast, Tokyo, Japan) and free-drinking either of oxygen-nanobubble distilled water or of normal distilled water. All distilled water was purchased from Otsuka Pharmaceutical Factory, Inc. (Tokyo, Japan). Oxygen-nanobubbles distilled water was filtered immediately after generation, and normal distilled water was filtered immediately after opening the lid, with 0.22 um pore size cellulose acetate membrane (Corning, Cambridge, MA) to avoid contamination. After filtration, DO was measured by handheld dissolved oxygen meter DO-24 P (DKK-TOA Corporation, Tokyo, Japan). Size (detection range; 10–1000 nm) and concentration of nanoparticles were measured by NanoSight LM10-HSBT14 (NanoSight Ltd, Salisbury, UK). Water was kept in 4°C for 12 weeks and sufficiently supplied in water bottles of mice twice a week after restoring to room temperature. All mice were purchased from CLEA Japan (Tokyo, Japan), and housed in a room under controlled temperature (23±1°C) and humidity (45–65%).

### Statistical Analysis

Data are expressed as mean ± standard error (SE) in the growth of Brassica campestris. Other data are expressed as mean ± standard deviation (SD). Differences between groups were assessed by Student's t-test. Any P value<0.05 was considered statistically significant.

## Results

**Figure 2:**
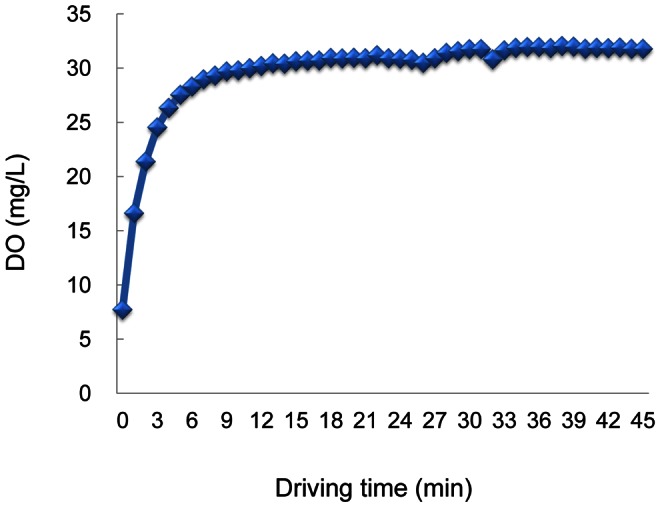
Sequential dissolved oxygen concentration (DO) in oxygen-nanobubble mixed water. Oxygen-nanobubbles were generated by the same methods as Figure 1 with 100 L water. Oxygen concentration was measured sequentially by Winkler's method.

**Figure 3:**
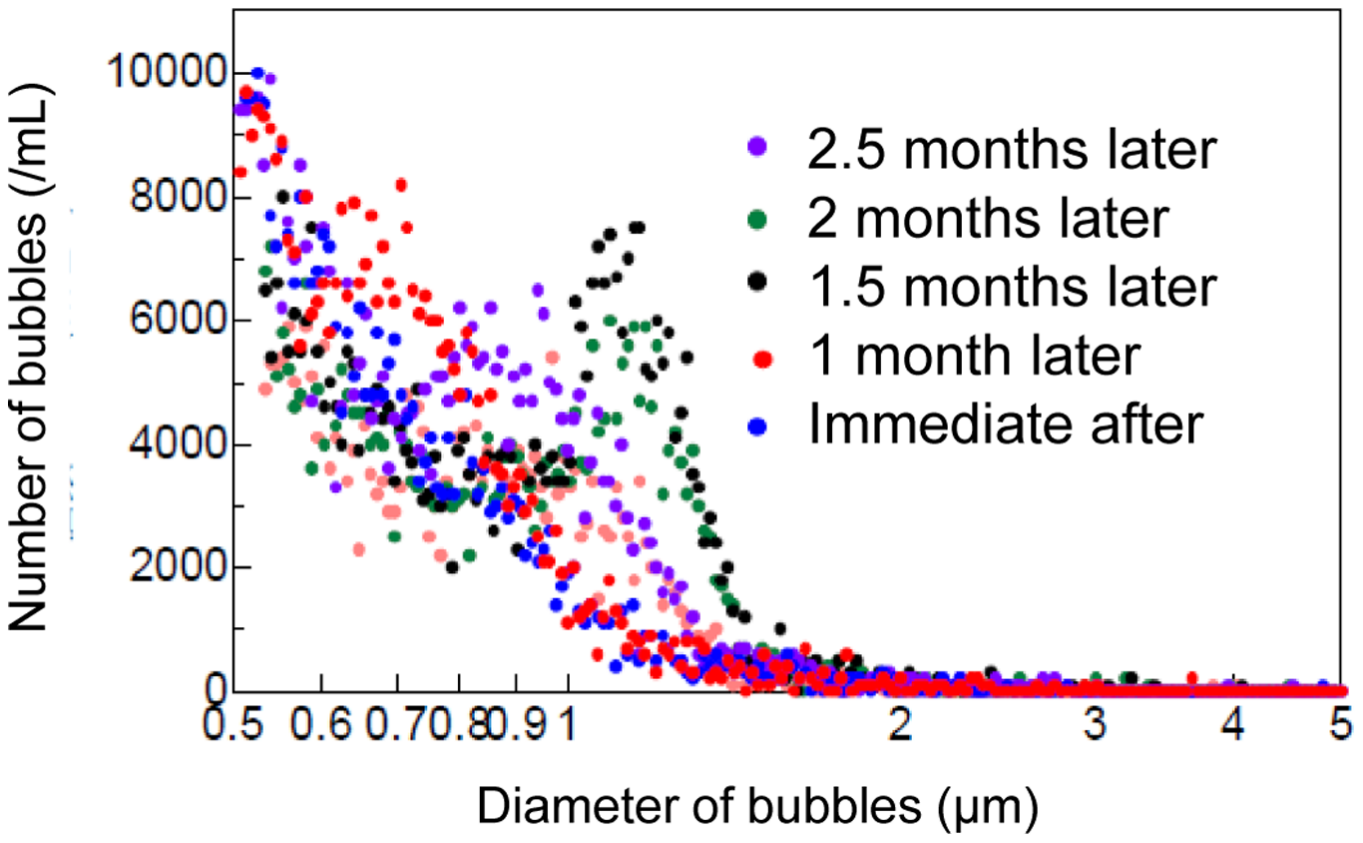
Sequential changes of number and diameter of generated air-nanobubbles. After generating air-nanobubble water by the same methods as Figure 1, sequential changes of number and diameter of generated air-nanobubbles were analyzed by Multisizer 3 (Beckman Coulter, Inc., Miami, FL, USA).

Oxygen concentration was 7.7 mg/L in original normal distilled water, whereas it increased to 31.7 mg/L in oxygen-nanobubble water immediately after running nanobubble aerator with 100 L water for 30 min (Figure 2). Figure 3 shows the chronological change and distribution of number and diameter of air-bubbles in water after generation. Approximately 70% of the generated air-bubbles were smaller than 2 µm in diameter immediately after generation. Moreover, even 2.5 months after generation, approximately 60% still remained smaller than 2 µm in diameter.

**Figure 4:**
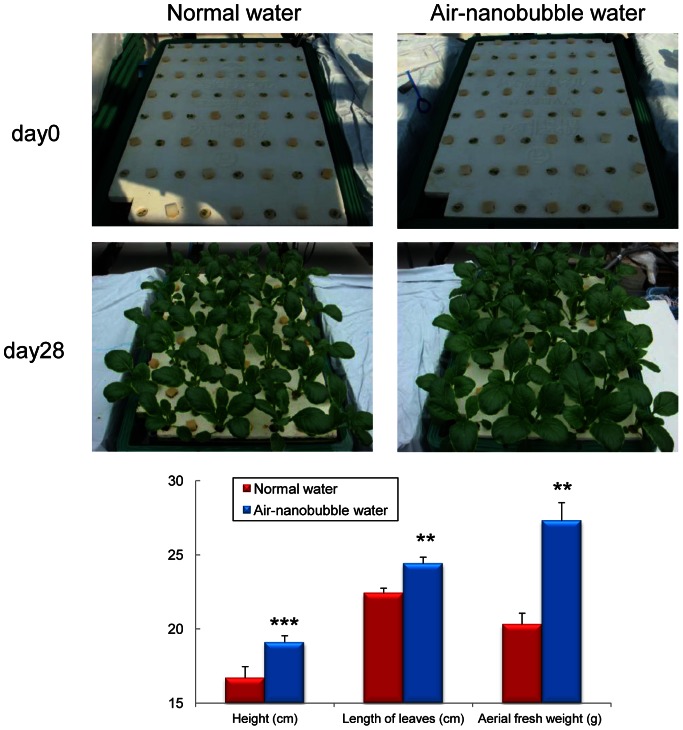
Growth of Brassica campestris cultured with either normal water or air-nanobubble water for 4 weeks. Brassica campestris (32 stumps in each group) were hydroponically cultured for 4 weeks. Water was circulated (38 L/min) in a tank (125 L/tank) and air-nanobubble solution was continuously generated using the nanobubble aerator. The water level of the tank and water temperature (from 23.2 to 24.3°C) was maintained throughout the culture. Data are shown as mean ± S.E. (n = 32 in each group). **P<0.01; ***P<0.001.

**Figure 5:**
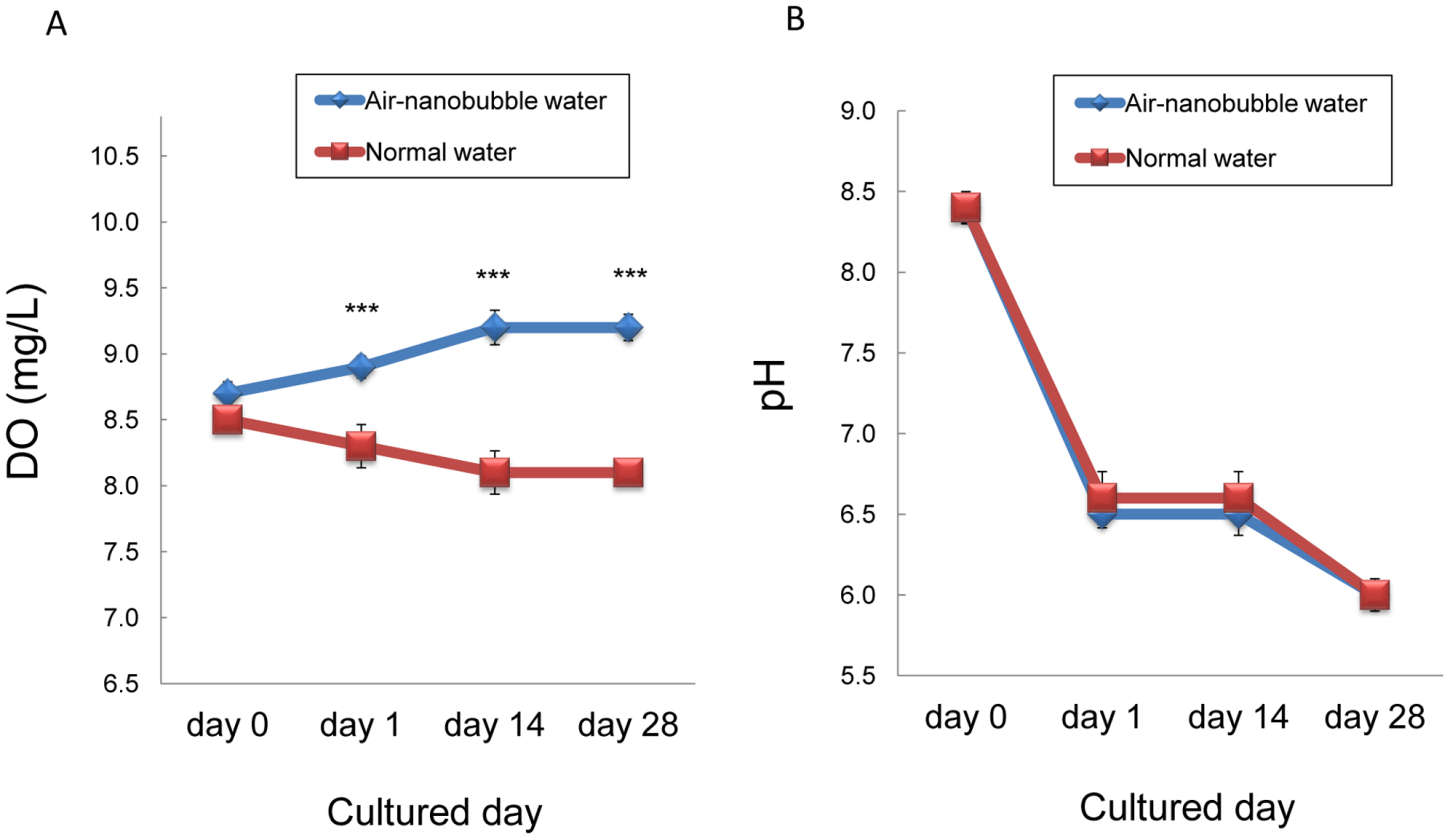
Sequential changes of dissolved oxygen concentration (DO) and pH of cultured water. DO (A) and pH (B) of Brassica campestris cultured water were monitored sequentially. Data are shown as mean ± S.D. (n = 5 in each group). ***P<0.001.

Air-nanobubble water significantly promoted the height (16.7±2.0 vs. 19.1±2.5 cm; P<0.001), length of leaves (22.4±4.3 vs. 24.4±2.5 cm; P<0.01), and aerial fresh weight (20.3±4.3 vs. 27.3±6.9 g; P<0.01) of Brassica campestris compared to normal water after hydroponical culture for 4 weeks (Figure 4). DO of cultured water was significantly higher (P<0.001) in air-nanobubble water compared to normal water from day 1 to day 28 (Figure 5A). On the other hand, there was no significant difference in pH (Figure 5B), nitrogen, phosphorus, potassium, calcium, and magnesium concentration within cultured water throughout the cultured period between two groups (data not shown).

After 3 weeks, total weight of sweetfish increased from 3.0 to 6.4 kg in normal water, whereas it increased from 3.0 to 10.2 kg in air-nanobubble water (Table 1). In addition, total weight of rainbow trout increased from 50.0 to 129.5 kg in normal water, whereas it increased from 50.0 to 148.0 kg in air-nanobubble water after 6 weeks (Table 1).

**Table 1:** Change in total weight of fishes after keeping within normal water or air-nanobubble water.

	Keeping period		Normal Water group	Air-nanobubble water group
Sweetfish	3 weeks	Initial total weight, kg	3.0	3.0
		Final total weight, kg	6.4	10.2
Rainbow trout	6 weeks	Initial total weight, kg	50.0	50.0
		Final total weight, kg	129.5	148.0

**Figure 6:**
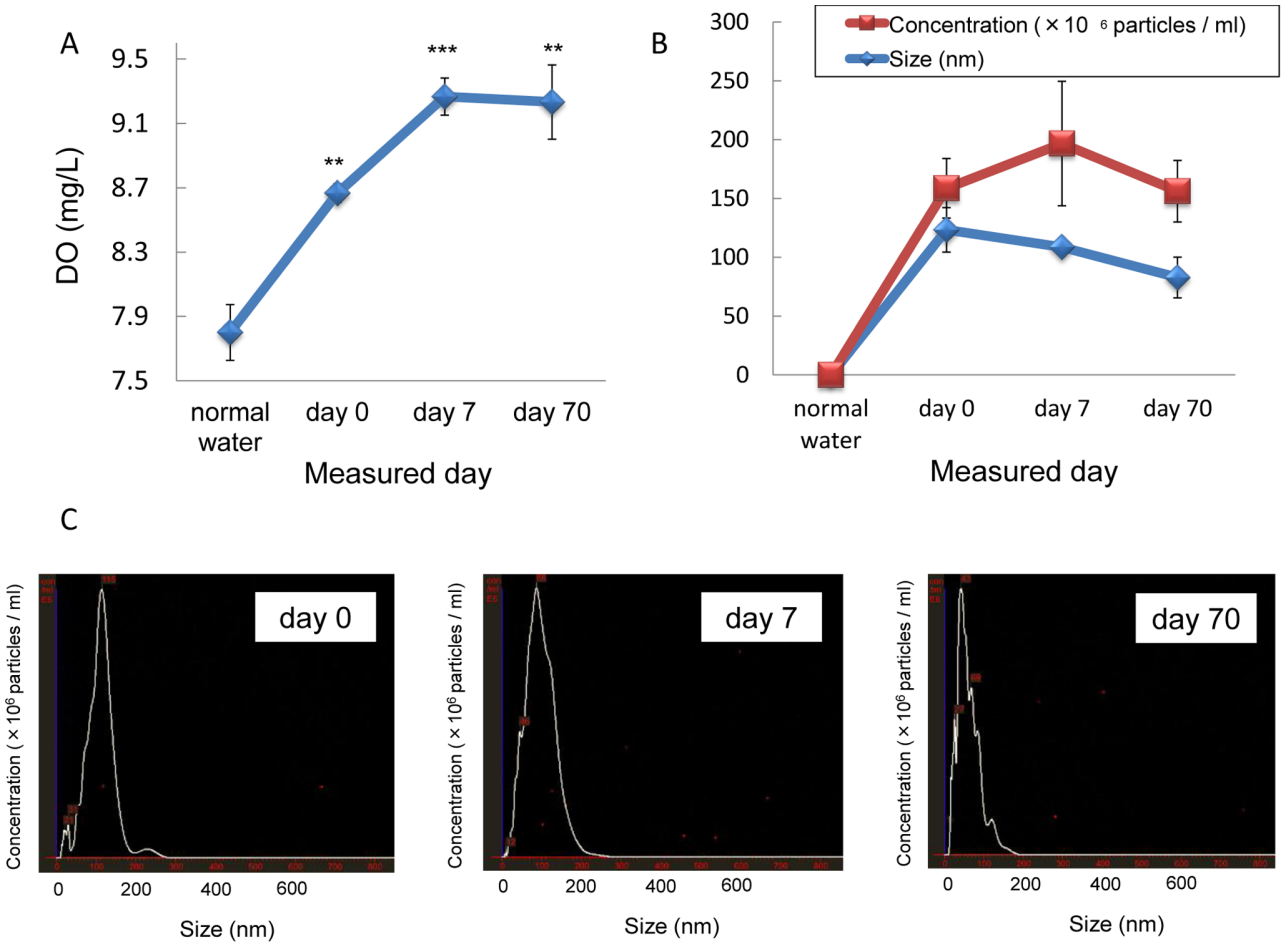
Sequential changes of dissolved oxygen concentration (DO) and concentration/size of oxygen-nanobubble distilled water from day 0 to 70. DO (A) and concentration/size (B) of oxygen-nanobubble distilled water were monitored sequentially. Distribution of concentration and size of nanoparticles are shown (C). Data are shown as mean ± S.D. (n = 3 in each group). **P<0.01; ***P<0.001 compared to normal water.

In our preliminary experiments, hyperoxidation as much as about 31.7 mg/L of DO in oxygen-nanobubble water took place immediately after generation (Figure 2), but it fell to a constant level about 8.7 mg/L at 5 hr after generation and then maintained from day 7 to 70 (Figure 6 A). Nanoparticles were not detected in normal distilled water initially, but were clearly detected after generation of oxygen-nanobubble. The concentration of nanoparticles was maximum at day 7 and mostly maintained at day 70, while the size of nanoparticles decreased gradually but maintained as long as day 70 (Figure 6 B and C). DO of oxygen-nanobubble distilled water did not change before and after filtration (data not shown).

**Figure 7:**
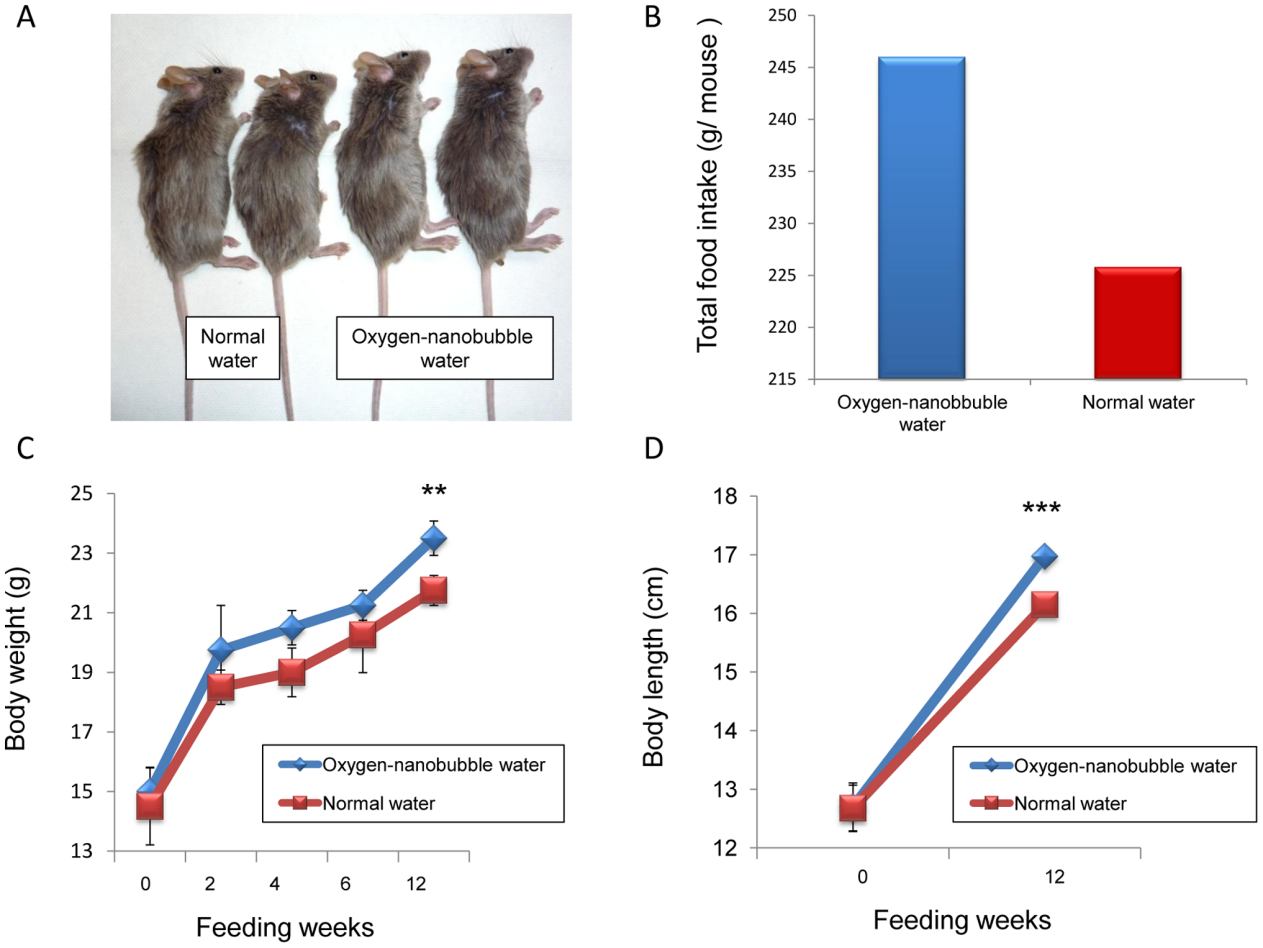
Sequential changes of food intake, body weight, and body length of mice. 5 weeks old male DBA1/J mice were bred with normal free-chaw and free oral intake either of oxygen-nanobubble distilled water or of normal distilled water for 12 weeks (A). Total food intake (B), body weight (C), and body length (D) were monitored in each mouse. Data are shown as mean ± S.D. (n = 4 in each group). **P<0.01; ***P<0.001.

After 12 weeks, free oral intake of oxygen-nanobubble distilled water significantly promoted the weight (21.8±0.3 vs. 23.5±0.3 g; P<0.01) and the length (16.1±0.1 vs. 17.0±0.1 cm; P<0.001) of mice as compared to free oral intake of normal water (Figure 7 A, C, D). As for food consumption, mice drinking oxygen-nanobubble water took higher dose of food compared to that of normal water (Figure 7 B).

## Discussion

To the best of our knowledge, we demonstrated for the first time that air and oxygen-nanobubbles promote the growth of plants, fishes, and mice. Yoshida et al. demonstrated that cucumber plants at higher dissolved oxygen concentration showed increased area and weight of leaves [13]. In addition, Park et al demonstrated that air-microbubbles promote the growth of leaf lettuce compared to air-macrobubbles, in which report DO of cultured water at the beginning was very similar to that of us (about 8.5 mg/L) [15]. On the other hand, DO in cultured water on day 14 was relatively higher in our study (air-nanobubble water; about 9.2 mg/L) as compared to Park’s report (air-microbubble water; about 8.2 mg/L). This difference is in accordance with the previous report which speculated nannobubbles increase the oxygen partial pressure (PO_2_) values in water to greater extent than that of microbubbles [1], [2]. In our study, nutrient solution except for oxygen did not differ between two groups (data not shown), but Park et al also speculated that larger specific surface area of the microbubbles as well as negative electronic charges on their surface may promote the growth of plants because microbubbles can attract positively charged ions that are dissolved in the nutrient solution. Regarding vertebrates, Owerkowicz et al. demonstrated that the growth of American alligators was the fastest when maintained at hyperoxia, while it was the slowest at hypoxia [14]. They suggested that combination of elevated metabolism and low demand of breathing in hyperoxic condition allows a greater proportion of metabolized energy to be available for growth. They also suggested that hyperoxia may induce hypermetabolic state to maintain higher rate of food digestion and absorption. These reports are in accordance with the results of our study, suggesting that air and oxygen-nanobubble water solution may contribute to elevated metabolism, higher food intake, and promoted growth.

In this study, there were no obvious abnormalities or harmful effects in fishes and mice with oral intake of air and oxygen-nanobubbles, respectively. No morphological irregularity was recongnized in Brassica campestris cultured with air-nanobubble water, either. It has also been suggested that nanobubbles could be clinically safe in human; because the filter pore size of cardiopulmonary bypass machines are usually in the range of 28–40 µm, nanobubbles of <10 µm in diameter are therefore negligibly small as a causal substance of gas embolism in blood vessels [18]. Cavalli et al also reported that fluorescent labeled nanobubbles showed good capacity of loading oxygen without hemolytic activity or toxic effect in Vero cells [19]. Taken together, it may be said that air and oxygen-nanobubbles could be safe accelerator of growth in most of lives. In addition, DO as well as concentration/size of nanoparticles in oxygen-nanobubble distilled water were relatively stable in 4°C from day 0 to 70.

There are several limitations in this study. In fishes and mice experiment, food intake was not controlled. The promoting effect of air and oxygen-nanobubble water on growth may be partially due to increase in oral uptake. In addition, although previous study have demonstrated that oxygen-nanobubble saline effectively improved hypoxic conditions of swine blood ex vivo [2], actual Po_2_ in blood of fishes and mice are not monitored. As we have not used other gas-nonobubble water, whether promoting effect on growth is due to nanobubbles themselves or elevated oxygen concentration in the water is still unresolved. Further examination using other gas nanobubbles is required to determine the effect of nanobubbles themselves on growth of lives. Although there are several limitations, air and oxygen-nanobubble water significantly promoted the growth of plants, fishes, and mice, and this novel finding may bring new insight in effective growth of lives in the future.
